# Revision Surgery for Postoperative Spondylodiscitis at Cage Level after Posterior Instrumented Fusion in the Lumbar Spine—Anterior Approach Is Not Absolutely Indicated

**DOI:** 10.3390/jcm9123833

**Published:** 2020-11-26

**Authors:** Jen-Chung Liao, Wen-Jer Chen

**Affiliations:** Department of Orthopedic Surgery, Bone and Joint Research Center, Chang Gung Memorial Hospital, Chang Gung University, No.5, Fu-Shin Street, Kweishian, Taoyuan 33302, Taiwan; chenwj@adm.cgmh.org.tw

**Keywords:** interbody fusion cage, spondylodiscitis, revision surgery

## Abstract

Spondylodiscitis at the cage level is rare but remains a challenge for spine surgeons. In this study, the safety and efficacy of revision surgery by a posterior approach to spondylodiscitis developed at the cage level were evaluated, and these data were compared to those of patients treated with revision surgeries using the traditional anterior plus posterior approach for their infections. Twenty-eight patients with postoperative spondylodiscitis underwent revision surgeries to salvage their infections, including 15 patients in the study group (posterior only) and 13 patients in the control group (combined anterior and posterior). *Staphylococcus aureus* was the most common pathogen in both groups. L4-L5 was the most common infection site in both groups. The operation time (229.5 vs. 449.5 min, *p* < 0.001) and blood loss (427.7 vs. 1106.9 mL, *p* < 0.001) were the only two data points that were statistically significantly different between the two groups. In conclusion, a single posterior approach with ipsilateral or contralateral transforaminal lumbar interbody debridement and fusion plus extending instrumentation was safe and effective for spondylodiscitis developed at the cage level. This strategy can decrease the operation time and blood loss.

## 1. Introduction

Postoperative surgical site infection in spinal surgery may be a well-known complication, but remains a challenge for patients and the treating surgeon. The incidence of postoperative spinal infections varies widely from 1% to 25% in different procedures and in patients with underlying diseases [1,2,3]. Most cases of early postoperative spinal infections are often managed successfully by aggressive debridement and culture-guided antibiotics, even in patients with spinal instrumentation [4,5]. Removal of the implant is suggested when the infection occurs after fusion is completed and there is no concern of spinal instability [6,7]. The most challenging cases for spinal surgeons include patients with infection developed anteriorly after posterior procedures. Anterior debridement and fusion followed by an extra posterior fixation are rationally recommended for spondylodiscitis after posterior instrumented fusion [8]. However, infective spondylodiscitis can be surgically treated through the posterior transforaminal approach, and clinical results have also shown that it is feasible for lumbar spondylodiscitis [9]. We hypothesized that lumbar spondylodiscitis developed at the cage level can be managed successfully by the posterior approach only, and the anterior retroperitoneal approach is not always necessary for this type of infection. The purposes of this study were to determine the safety and efficacy of revision surgery using a posterior approach to spondylodiscitis developed at the previous cage level, and to compare these data with those of patients treated with revision surgeries using the traditional anterior plus posterior approach for their infections. 

## 2. Materials and Methods

This study was performed after obtaining approval from the institutional review board of the Chang Gung Memorial Hospital (No. 202001630B0). From 2006 to 2017, 7260 patients with degenerative lumbar or thoracolumbar spinal diseases who underwent posterior instrumentation with pedicle screws and posterior transforaminal interbody fusion with fusion cages were reviewed retrospectively. Postoperative spinal infection was found in 167 patients (2.3%). Postoperative spondylodiscitis was noted in 28 patients who underwent revision surgeries in order to salvage their anterior infections (0.38%). Postoperative spondylodiscitis was confirmed by clinical symptoms and magnetic resonance imaging findings including a hypointense signal on T1-weighted images, a hyperintense signal on T2-weighted images, ring-enhancement around the disc space of the infected cage, or paraspinal abscess formation after contrast infusion. The indications for revision surgeries included failure of antibiotic treatment, intractable pain, and/or radicular pain due to spinal instability or cage dislodgement. Before 2013, all cases of postoperative spondylodiscitis of the cage were surgically treated with combined anterior and posterior approaches. Inspired by the method of Lu et al. [8], we started to use a single posterior approach to surgically manage postoperative spondylodiscitis in 2014. 

This was a two-cohort comparison study. According to the approaches to their infection sites, these 28 patients were divided into the study group (posterior only) and the control group (combined anterior and posterior). Several parameters in each group were recorded, including age, sex, elapsed time (primary surgery to index revision surgery), initial treated segments, level of loosening pedicle screw, and level of infected cage. Laboratory data included white blood cell (WBC) count, erythrocyte sedimentation rate (ESR), C-reactive protein (CRP), and cultured pathogen for infection. Stages of anesthesia by American Society of Anesthesiologists (ASA), underlying diseases recorded using the Charlson Comorbidity Index (CCI), and body mass index (BMI) of each patient were assessed. To compare the efficacy of each surgical strategy, parameters, including operation time, blood loss, positive culture rate, hospital stay, fusion status, and final clinical outcomes, were used for comparison. The patients were followed up at 3 months, 6 months, and 1 year after revision surgery, and then annually. The clinical outcomes were evaluated using the visual analog scale (VAS) and the Oswestry Disability Index (ODI). The fusion status was assessed using 1-year radiographs according to the Bridwell criteria [10]. 

### 2.1. Operative Procedure

The study group: the revision methods in this group were the posterior removal of loosening screws, extending instrumentation to inferior or superior healthy vertebrae; the infected cage was removed as much as possible, and the infected disc space was debrided and fused with autologous iliac bone graft through the ipsilateral or contralateral foramina tunnel. 

The control group included patients who underwent traditional anterior debridement, removal of the infected cage, interbody fusion with a structured tricortical bone graft, and simultaneous or staged posterior surgery for revision of posterior instrumentation. Whether the patient received simultaneous or staged surgery depended on the patient’s condition and the facility of the operation room available at the time. 

After revision surgery, all patients were encouraged to sit upright on the second postoperative days and began lower limb exercises to prepare for ambulation. All patients were instructed to wear a Taylor brace for 3 months. 

### 2.2. Antibiotic Treatments 

Antibiotic were withheld until culture data was obtained. The appropriate antibiotics, as determined by culture results, were typically intravenously administrated for 4 weeks, followed by 4 weeks of oral antibiotics. The patient with tuberculosis infection received a combination of isoniazid, rifampin, ethambutol, and pyrazinamide for 9 months. The patient with fungus infection were treated with fluconazole and amphotericin B for 12 weeks.

### 2.3. Statistical Analysis 

The Chi-square test and Fisher’s exact test were used for categorical variables. The Mann–Whitney U test was used for continuous variables. A two-tailed value of *p* < 0.05 was considered statistically significant.

## 3. Results

A total of 28 patients were enrolled in this study: 12 women and 16 men. The mean age at revision surgery was 66.8 years (range, 58–78 years). Postoperative spondylodiscitis at the cage level was diagnosed between 0.5 and 11 months after primary posterior surgery. The main symptom of these 28 patients was recurrent intractable back pain, and 7 patients had radicular pain (25%). The average VAS score for back pain was 7.9 (range, 7–9). The WBC count was normal or slightly elevated (mean: 8728/mm^3^). The mean ESR level was 77.4 mm/h (range, 10–119 mm/h). The mean CRP level was 79.2 mg/L (range, 2.8–218 mg/L). L4-L5 was the most common infection site (20 patients), followed by L5-S1 (3 patients), L3-L5 (1 patient), L4-S1 (1 patient), L3-4, (1 patient), L2-3 (1 patient), and L1-2 (1 patient). Underlying diseases included diabetes mellitus, hypertension, end-stage renal disease, rheumatoid arthritis with steroid treatment, Parkinson’s disease, coronary heart disease, and cancer history. Of the 28 patients, 24 had positive culture pathogens, of which 22 had bacterial infections; one patient had *Mycobacterium tuberculosis*, and one patient had *Candida albicans*. Bacterial infections included *Staphylococcus aureus*, *Staphylococcus epidermidis*, *Staphylococcus warneri*, *Staphylococcus lugdunensis*, *E. coli*, *Propionibacterium acnes*, *Streptococus viridans*, *Pseudomonas stutzeri*, Propionibacterium species, and *Klebsiella pneumoniae*. None of the patients had neurologic deterioration due to this revision surgery. Loosening pedicle screws were found in all 28 patients, especially in the inferior vertebrae of the infected cage (for example, L4-5 cage infection and L5 pedicle screws). Even after revision surgery, four patients still had some degree of posterior surgical wound infection requiring surgical debridement. All patients demonstrated complete eradication of infection. Based on the Bridewell anterior fusion grading system, 14 patients (50%) achieved grade I fusion, 12 patients (43%) achieved grade II fusion, one patient (3.6%) achieved grade III fusion, and one patient (3.6%) achieved grade IV fusion. Clinically, the mean VAS score for back pain improved from 7.9 preoperatively to 3.3 finally. The mean ODI score improved from 46 to 18.5. The data of these 28 patients are summarized in Table 1.

**Figure 1:**
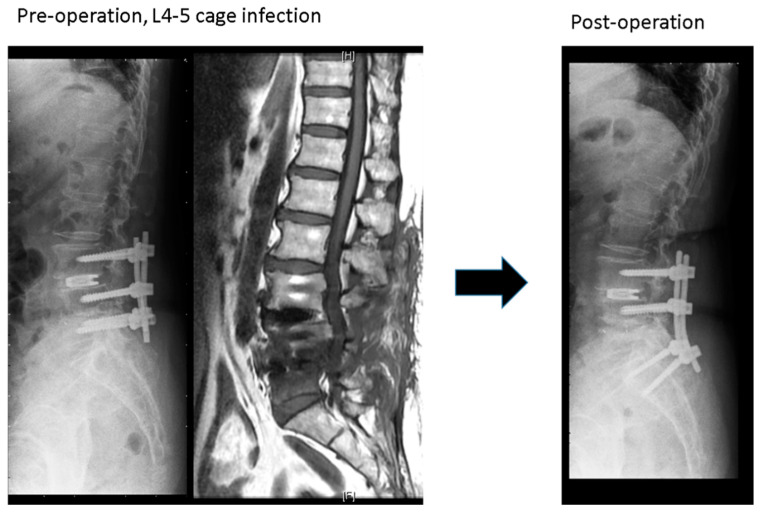
A case in the study group: L4-5 cage infection treated by removal of the L5 screws, extending screw to S1 with contralateral transforaminal debridement and fusion at L4-5 disc space.

The study group (Figure 1) included 15 patients: 8 women and 7 men with a mean age of 67.2 years. The mean BMI, CCI, and ASA were 25. 4 ± 2.6 kg/m^2^, 1.8 ± 0.8 and 2.9 ± 0.4, respectively. The mean interval between primary and revision surgeries was 91.5 ± 76.3 days. The L4-5 segment was the most common infection site (10 patients, 66%). The mean operation time and blood loss were 229.5 ± 37.3 min and 427.7 ± 250.3 mL, respectively. Thirteen patients in the study group had positive culture data; *Staphylococcus aureus* and *Staphylococcus epidermidis* were the most common pathogens. Eight patients’ infected cages could be removed successfully, but the other seven patients’ cages were retained at where left they were. All these 15 patients’ infections were cured after surgery and intravenous antibiotic treatment, with an average hospital stay of 28 days. Before revision surgery, the mean VAS score was 8.0 ± 0.7, which finally improved to 3.5 ± 1.2. The ODI also improved from 44.2 ± 11.5 preoperatively to 18.5 ± 12.2 finally. The final radiographs showed that six patients achieved grade I fusion, eight patients were assessed as grade II fusion, and one patient’s fusion status was grade IV.

**Figure 2:**
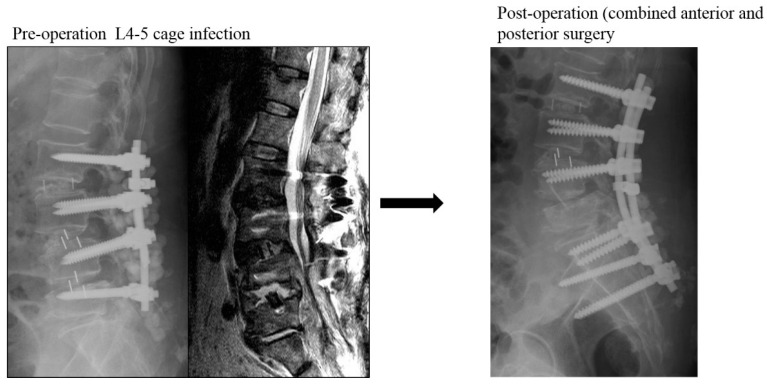
A case within the control group: L4-5 cage infection treated by anterior removal of cage and fusion, followed by posteriorly removal of L5 and extending instrumentation to S1 and ilium.

The other 13 patients were grouped into the control group (Figure 2), including 4 women and 9 men with a mean age of 66.6 years at surgery. All patients in this group underwent revision surgeries by simultaneously or staged anterior combining posterior surgery for infectious spondylodiscitis. All infected cages were removed, and reconstructions were performed with autologous iliac tricortical bone grafts or allogenic cortical bone grafts. The mean BMI, CCI, and ASA were 26/8 ± 3.4 kg/m^2^, 2.0 ± 1.2, and 3.0 ± 0.0, respectively. The average interval between primary and revision surgery was 99.4 ± 84.7 days. The L4-5 segment was still the most common infection site (10/13). The total sum of anterior and posterior revision surgery mean operation time and blood loss were 449.5 ± 91.3 min and 1106.9 ± 536.5 mL, respectively. Eleven patients had positive culture data, and Staphylococcus species (seven cases) remained the most common pathogens. The average hospital stay was 32.6 days, and all patients were doing well by the final follow-up. The mean VAS score was 7.8 ± 0.8 before revision surgery and improved to 3.0 ± 1.1 at the last follow-up. Finally, the ODI also improved from 48.1 ± 8.7 preoperatively to 18.5 ± 14.1. There were three patients with poor healing of posterior wounds, including one patient who needed removal of all posterior instrumentations when the anterior infection segment was fused. The average anterior fusion scale score was 1.4: eight patients with grade I, four patients with grade II, and one patient with grade III. 

### Comparisons between the Two Groups

All preoperative demographic data, including age at surgery, sex ratio, BMI, CCI, ASA, and previous surgical segments, were similar. In both groups, the positive culture rates were similar and the L4-5 segment was the most common revision site. The positive culture rate was also similar using both surgical methods. The study group had a shorter hospital stay during revision surgery, but with no statistically significant difference. According to the ODI and VAS scores, clinical outcomes were not significantly different between the two groups. The control group seemed to have a better radiographic score (1.4 vs. 1.7, *p* = 0.387) but still without a statistically significant difference. The operation time and blood loss were the only two parameters with a statistically significant difference between the two groups: the study group had a shorter operation time and lesser surgical blood loss. All comparisons between the two groups are listed in Table 2. 

## 4. Discussion

Postoperative spinal infection is a relatively common complication and sometimes a devastating consequence of spinal surgery. Wide exposure, prolonged operation time, and comorbidity are risk factors for postoperative spinal infection. Most cases of superficial or deep wound infection are often managed successfully by wound debridement, irrigation, and proper antibiotic treatment. The incidence of spondylodiscitis induced by the posterior spinal instrumentation procedure without a disc procedure is comparatively low. Hsieh et al. reported their experiences with the management of 11 cases of spondylodiscitis out of 6120 posterior instrumented posterolateral fusions (0.18%); the anterior infections were successfully treated with a combination of anterior debridement/fusion and posterior instrumentation [8]. In order to increase the fusion rate, correct deformity, and acquire a better lumbar lordosis, interbody fusion cages have become popular in the last 20 years. When compared to posterior instrumented posterolateral fusion, the posterior instrumented interbody fusion procedure might increase operation time and blood loss, which could increase the incidence of spondylodiscitis. This spondylodiscitis was believed to have spread from the posterior infection via a pedicle screw or cage. During this study, 28 of 7268 patients (0.38%) experienced spondylodiscitis after posterior instrumented surgeries; the incidence was twice that reported by Hsieh et al. Ahn et al. also demonstrated that the infection rate of instrumented posterior interbody fusion (PLIF) was fourfold that of instrumented posterolateral fusion (PLF) (1.37% vs. 0.3%); 67% in PLIF infection and 33% in PLF infection had osteomyelitis [11].

There has been no consensus on the conclusions regarding the management of a postoperatively infected interbody cage from previous studies. Ha et al. were the first to successfully describe the surgical treatment of postoperative spondylitis that occurred after posterior lumbar interbody fusion using cages [12]. They reported their 10 cases and suggested anterior removal of the infected cage and concomitant removal of the posterior instrumentation to salvage spondylitis. Carmouche et al. reported a case and recommended the removal of the cage by the anterior approach, with removal of posterior pedicle instrumentation to control infectious discitis [13]. In contrast, Mirovsky et al. reported that posterior debridement, cage repositioning, and proper intravenous antibiotic treatment could successfully control osteomyelitis around cages. They concluded that removal of the interbody implant was not necessary for an infected cage [14]. Lee et al. reported revision methods for 10 cases with organ/space infection after posterior instrumented interbody fusions. Nine cases achieved infection control by posterior one-stage simultaneous revision, which involves removal of the cage, irrigation of the infected disc space, bone graft in the interbody space, and exchange of the larger pedicle instrumentation simultaneously using the posterior approach only. The other case was treated with anterior cage removal and anterior interbody fusion with a bone graft while preserving posterior instrumentation [15]. Our experiences were similar to those of Lee at al. The infected cages were successfully removed, the disc space was debrided, and bone grafts were inserted from a posterior approach in eight patients of the study group. The cages could not be removed in the remaining seven patients, but debridement of the infected disc space and insertion of a bone graft were performed through a contralateral transforaminal approach. Chang et al. reported surgical results of 32 patients with infected transforaminal lumbar interbody fusion cages and compared radiographic and functional outcomes between cage removal and cage retention [16]. They suggested that infected cages should be removed because the cage-retained group had poorer outcomes. These 17 cases within the cage-retained group of Chang et al. were managed only by posterior debridement without additional procedures for augmentation. In contrast, extending instrumentation to the healthy vertebrae and/or exchanging pedicle screws of larger size by performing contralateral interbody fusion combined with posterolateral fusion were performed in our seven cases with retained cages. We believe that providing additional spinal stability and fusion were the most important keys for successful treatment of spondylitis in these cages-retained patients. 

Spontaneous spondylodiscitis involves the vertebrae and disc, which are located at the anterior part of the spinal unit. Traditionally, anterior debridement of infected materials followed by anterior or posterior fixation remains a reasonable method for surgical management of infectious spondylodiscitis [17,18,19]. The posterior approach alone for the treatment of infectious spondylodiscitis seems novel; only a few studies have reported this. Fushimi et al. reported six cases of pyogenic spondylitis that underwent posterior instrumentation and fusion alone without anterior debridement; the ultimate outcomes were satisfactory in these cases [20]. Lin et al. also demonstrated that their 48 cases of pyogenic spondylitis that underwent long-segment instrumentation with short fusion obtained significantly infection control [21]. They emphasized that adequate stability of spinal elements, achievement of posterolateral fusion, and proper antibiotic treatment were keys to achieve the successful management of spinal infections. The methods of transforaminal lumbar interbody fusion (TLIF) and posterior instrumentation for the surgical management of spinal tuberculosis were first described by Zaveri et al., and 87% of their cases (13/15) showed excellent or good final results [22]. Lu et al. modified the surgical procedure of TLIF and performed debridement, fusion, and instrumentation employing a single posterior approach (named TLIDF) for treating lumbar infectious spondylitis. This modified approach had a rate of infection clearance similar to that with the traditional combined anterior and posterior approach, and prevented kyphosis deformity [9]. In our study, 15 patients in the study group underwent TLIDF with extending posterior instrumentation for anterior spondylodiscitis. Although there were seven patients’ cages that could not be removed through the ipsilateral approach, contralateral TLIDF with additional posterolateral fusion was performed, and the final results were satisfactory. We therefore recommend posterior TLIDF with posterolateral fusion for postoperative spondylodiscitis, even in cases with infected cages. 

## 5. Conclusions

A posterior ipsilateral or contralateral TLIDF, with or without cage removal, combined with extension of posterior instrumentation is a safe and effective procedure to treat postoperative spondylodiscitis. The culture rate and final results of this procedure are similar to those of the combined anterior and posterior approach. This strategy can decrease the operation time and blood loss, and avoid the risks of anterior or staging surgeries. 

## Figures and Tables

**Table 1:** Demographic data of total 28 patients.

Characters	
Age (years)	66.8
Sex (M:F)	16:12
BMI	26.2
CCI	1.9
ASA	3
Surgical segments	3.1
Infected level	
L1-2	1
L2-3	1
L3-4	1
L3-5	1
L4-5	20
L4-S1	1
L5-S1	3
Interval between primary and revision surgery (days)	95.5
Laboratory	
WBC (X1000)(/mL)	8.7
ESR (mm/h)	77.4
CRP (mg/L)	79.2
Operation time (minutes)	331.6
Blood loss (mL)	743
Clinical outcomes	
Preoperative VAS	7.9
Postoperative VAS	3.3
Preoperative ODI	46
Postoperative ODI	18.5
Fusion score	
Grade 1	14
Grade 2	12
Grade 3	1
Grade 4	1
Pathogens	
*Staphylococcus aureus*	6
*Staphylococcus epidermidis*	5
*Staphylococcus warneri*	2
*Staphylococcus lugdunensis*	1
*E coli*	2
*Propionibacterium acnes*	1
*Streptococcus viridans*	1
*Pseudomonas stutzeri*	1
*Propionibacterium sp.*	2
*Klebsiella pneumoniae*	1
*Mycobacterium tuberculosis*	1
*Candida albicans*	1
No growth	4

Abbreviations: N = number, M = male, F = female, BMI = Body Mass Index, CCI = Charlson Comorbidity Index, ASA = American Stage of Anesthesia, WBC = white blood-cell count, ESR = erythrocyte sedimentation rate, CRP = C-reaction protein, VAS= visual analog scale, ODI = Oswestry Disability Index.

**Table 2:** Comparisons between the study and the control group.

Characters	Study Group (*N* = 15)	Control Group (*N* = 13)	*p* Value
Age	67.0 ± 5.8	66.6 ± 6.9	0.856
Sex (M:F)	7:08	9:04	0.229
BMI	25. 4 ± 2.6	26.8 ± 3.4	0.363
CCI	1.9 ± 0.8	2.0 ± 1.2	0.928
ASA	2.9 ± 0.4	3.0 ± 0.0	0.555
Surgical segments	2.9 ± 1.0	3.3 ± 1.9	
Infected level			
L1-2	0	1	
L2-3	1	0	
L3-4	1	0	
L3-5	0	1	0.246
L4-5	10	10	
L4-S1	0	1	
L5-S1	3	0	
Interval between primary and revision surgery (days)			
	91.5 ± 76.3	99.4 ± 84.7	0.13
Laboratory			
WBC (X1000) (/mL)	8.2 ± 2.4	9.9 ± 3.4	0.17
ESR (mm/h)	76.5 ± 8.2	78.3 ± 32.1	0.555
CRP (mg/L)	81.0 ± 59.6	77.2 ± 49.9	0.859
Operation time (minutes)	229.5 ± 37.3	449.5 ± 91.3	<0.001
Blood loss (mL)	427.7 ±250.3	1106.9 ± 536.5	<0.001
Clinical outcomes			
Preoperative VAS	8.0 ± 0.7	7.8 ± 0.8	0.586
Postoperative VAS	3.5 ± 1.2	3.0 ± 1.1	0.274
Preoperative ODI	44.2 ± 11.5	48.1 ± 8.7	0.235
Postoperative ODI	18.5 ± 12.2	18.5 ± 14.1	0.786
Fusion score	1.7 ± 0.8	1.5 ± 0.7	0.387
Positive culture (*N*) (%)	13 (87%)	11 (85%)	0.677
Complications (*N*) (%)	3 (20%)	3 (23%)	0.843

Abbreviations: N = number, M = male, F = female, BMI = Body Mass Index, CCI = Charlson Comorbidity Index, ASA = American Stage of Anesthesia, WBC = white blood-cell count, ESR = erythrocyte sedimentation rate, CRP = C-reaction protein, VAS = visual analog scale, ODI = Oswestry Disability Index.
